# Pharmacokinetics, Safety, and Tolerability of Cefiderocol, a Novel Siderophore Cephalosporin for Gram-Negative Bacteria, in Healthy Subjects

**DOI:** 10.1128/AAC.02163-17

**Published:** 2018-02-23

**Authors:** Yutaka Saisho, Takayuki Katsube, Scott White, Hiroyuki Fukase, Jingoro Shimada

**Affiliations:** a Medical Affairs Department, Shionogi & Co., Ltd., Osaka, Japan; b Clinical Research Department, Shionogi & Co., Ltd., Osaka, Japan; c Glaxo SmithKline, Philadelphia, Pennsylvania, USA; d CPC Clinical Trial Hospital, Kagoshima, Japan; e Shionogi & Co., Ltd., Tokyo, Japan

**Keywords:** cefiderocol, pharmacokinetics, cephalosporin, Gram-negative bacteria, siderophores

## Abstract

Cefiderocol is a novel parenteral siderophore cephalosporin that shows potent efficacy against various Gram-negative bacteria, including carbapenem-resistant strains, *in vitro* and in preclinical models of infection. The aim of the present study was to evaluate the pharmacokinetics (PK), safety, and tolerability of cefiderocol after both single and multiple dosing by intravenous infusion over 60 min in healthy adult subjects. A single-ascending-dose study at doses of 100, 250, 500, 1,000, and 2,000 mg was conducted in 40 healthy Japanese males and females (6 individuals receiving the active drug and 2 individuals receiving a placebo per cohort). A multiple-ascending-dose study at doses of 1,000 (two groups) and 2,000 mg every 8 h (q8h) was conducted in 30 healthy Japanese and Caucasian males (8 individuals receiving the active drug and 2 individuals receiving a placebo per cohort). There were no serious or clinically significant adverse events (AEs) observed in either study. A single subject receiving 1,000 mg cefiderocol q8h was withdrawn due to AEs. Dose-proportional increases in the maximum plasma concentration (*C*_max_), the area under the concentration-time curve (AUC) from time zero to the time of the last quantifiable concentration after dosing, and the area under the concentration-time curve extrapolated from time zero to infinity were observed across the dose range of 100 to 2,000 mg. The mean plasma half-life of cefiderocol was 1.98 to 2.74 h. Cefiderocol was primarily excreted unchanged in the urine (61.5% to 68.4% of the dose). There was little accumulation of *C*_max_ and AUC by dosing q8h, and the PK of cefiderocol did not change with multiple dosing. This study indicates that single and multiple intravenous doses of cefiderocol at up to 2,000 mg are well tolerated in healthy subjects and exhibit linear PK at doses up to 2,000 mg.

## INTRODUCTION

There is an urgent need to develop new antibiotics to combat the recent worldwide increase in the incidence in multidrug-resistant Gram-negative bacterial strains (1). In particular, it is becoming more challenging to treat serious nosocomial infections caused by Gram-negative pathogens, such as Pseudomonas aeruginosa and Acinetobacter baumannii, as well as carbapenem-resistant Enterobacteriaceae (2). The 2017 global priority pathogens list from the World Health Organization has carbapenem-resistant A. baumannii and P. aeruginosa and carbapenem-resistant, third-generation cephalosporin-resistant Enterobacteriaceae as the three pathogens with the highest priority for the research and development of new antibiotics (3). The mechanisms responsible for carbapenem resistance in Gram-negative bacteria include the spread of exogenous carbapenemases (4, 5), overexpression of efflux pumps, overexpression of chromosomal β-lactamases, and a deficiency in outer membrane porins (4, 6–8).

Cefiderocol (also known as S-649266) is a novel catechol-substituted siderophore cephalosporin with potent *in vitro* and *in vivo* activity against a variety of Gram-negative bacteria, including carbapenem-resistant Enterobacteriaceae, P. aeruginosa, and A. baumannii (9–11, 20). The catechol moiety, which is found at the three-position side chain, forms a chelating complex with ferric iron (12). This allows cefiderocol to function as a siderophore and enter bacterial cells through active transport via a Trojan horse mechanism that utilizes the bacterial iron transport system (12). In an *in vitro* study in P. aeruginosa, the iron-mediated uptake of cefiderocol was shown to contribute to its antibacterial activity (12).

No siderophore antibiotics have been approved for clinical use to date, although naturally occurring and synthetic siderophore-conjugated antibiotics have been under investigation for several decades (13, 14). Cefiderocol is the first siderophore antibiotic to advance into late-stage development (15, 16). The primary objective of the present study was to evaluate the pharmacokinetics (PK), safety, and tolerability of single- and multiple-dose administration of cefiderocol by intravenous infusion in healthy Japanese and Caucasian adult subjects. A secondary objective was to evaluate the production of any cefiderocol metabolites.

## RESULTS

### Subjects.

A total of 70 subjects were enrolled in the study (40 in the single-dose study, 30 in the multiple-dose study). Of these, 54 received cefiderocol and 16 received placebo. Table 1 shows the demographics and baseline characteristics of all dosed subjects. The data obtained from all subjects receiving cefiderocol were included in the PK analysis.

**TABLE 1:** Subject demographics and baseline characteristics^*a*^

Characteristic	Single-dose study						Multiple-dose study			
	Cefiderocol					Placebo (*n* = 10)	Cefiderocol			Placebo (*n* = 6)
	100 mg (*n* = 6)	250 mg (*n* = 6)	500 mg (*n* = 6)	1,000 mg (*n* = 6)	2,000 mg (*n* = 6)		1,000 mg 1st (*n* = 8)	1,000 mg 2nd (*n* = 8)	2,000 mg (*n* = 8)	
Age (yr)										
Mean	23.3	26.7	38.8	28.8	30.5	34.5	32.6	29.6	34.1	31.8
SD	2.5	3.7	16.5	4.1	14.7	14.1	8.1	6.0	10.1	8.0
Range	20–27	23–32	26–60	25–36	21–60	21–59	21–46	22–38	21–49	24–47
No. (%) of male subjects	6 (100)	6 (100)	4 (66.7)	6 (100)	5 (83.3)	8 (80.0)	8 (100)	8 (100)	8 (100)	6 (100)
No. (%) of subjects by race										
Japanese	6 (100)	6 (100)	6 (100)	6 (100)	6 (100)	10 (100)	6 (75.0)	7 (87.5)	6 (75.0)	4 (66.7)
Caucasian	0	0	0	0	0	0	2 (25.0)	1 (12.5)	2 (25.0)	2 (33.3)
Ht (cm)										
Mean	167.93	170.73	159.13	169.85	166.12	168.30	174.50	170.35	176.24	175.33
SD	6.51	2.55	5.01	6.42	4.44	6.21	4.83	4.39	7.26	6.41
Range	156.4–176.0	167.1–173.2	153.1–166.3	158.0–177.5	158.0–170.8	156.7–176.0	167.8–181.1	164.9–177.4	166.8–185.6	169.3–184.6
Wt (kg)										
Mean	60.43	64.28	54.88	65.47	59.08	63.95	69.81	63.44	69.78	70.72
SD	5.70	4.48	5.10	5.81	4.26	8.33	10.30	4.72	11.03	8.55
Range	54.5–71.0	57.7–69.3	45.1–59.4	58.1–73.2	52.1–62.9	55.0–77.0	59.9–90.4	55.5–67.9	57.3–91.0	59.3–83.1
BMI (kg/m^2^)										
Mean	21.47	22.03	21.63	22.70	21.42	22.54	22.89	21.90	22.39	23.00
SD	2.25	1.45	1.43	1.59	1.41	2.26	2.79	2.05	2.57	2.54
Range	18.9–24.6	20.0–23.7	19.2–22.8	20.2–24.8	19.1–22.9	18.7–24.9	19.3–27.6	19.1–24.6	18.9–26.8	20.7–27.6
CL_CR_ (ml/min)										
Mean	140.0	168.2	116.3	147.5	131.3	141.6	141.5	130.6	133.5	121.5
SD	16.2	13.2	28.6	20.1	29.6	30.8	22.6	17.1	23.0	11.9
Range	121–168	151–185	75–151	120–174	81–173	98–192	99–176	110–166	90–161	101–132
eGFR (ml/min/1.73 m^2^)										
Mean	116.5	132.2	104.5	112.5	110.2	111.6	106.9	104.9	101.4	91.0
SD	7.5	8.7	20.4	15.7	21.8	23.4	14.5	11.6	13.8	12.8
Range	108–127	123–146	82–133	98–137	76–142	74–142	77–125	89–126	80–117	75–110

a BMI, body mass index; CL_CR_, creatinine clearance; eGFR, estimated glomerular filtration rate.

### Safety and tolerability.

Cefiderocol was generally well tolerated in the single-dose and multiple-dose studies (Tables 2 and 3).

**TABLE 2:** Incidence of adverse events in the single-dose study^*a*^

AE by system organ class, preferred term	Cefiderocol						Placebo (*n* = 10)
	100 mg (*n* = 6)	250 mg (*n* = 6)	500 mg (*n* = 6)	1,000 mg (*n* = 6)	2,000 mg (*n* = 6)	Total (*n* = 30)	
Any adverse event	1 (1), 16.7	0	1 (2), 16.7	2 (4), 33.3	2 (3), 33.3	6 (10), 20.0	4 (5), 40.0
Gastrointestinal disorders	0	0	1 (2), 16.7	0	1 (1), 16.7	2 (3), 6.7	2 (2), 20.0
Diarrhea	0	0	1 (1), 16.7	0	1 (1), 16.7	2 (2), 6.7	1 (1), 10.0
Abdominal pain	0	0	1 (1), 16.7	0	0	1 (1), 3.3	0
Nausea	0	0	0	0	0	0	1 (1), 10.0
Investigations	1 (1), 16.7	0	0	1 (3), 16.7	1 (1), 16.7	3 (5), 10.0	2 (2), 20.0
Urine positive for white blood cells	0	0	0	1 (1), 16.7	0	1 (1), 3.3	1 (1), 10.0
Blood creatine phosphokinase level increased	0	0	0	1 (1), 16.7	0	1 (1), 3.3	0
Blood present in urine	0	0	0	1 (1), 16.7	0	1 (1), 3.3	0
Urine positive for red blood cells	1 (1), 16.7	0	0	0	0	1 (1), 3.3	0
White blood cell count increased	0	0	0	0	1 (1), 16.7	1 (1), 3.3	0
Blood glucose level increased	0	0	0	0	0	0	1 (1), 10.0
Nervous system disorders	0	0	0	0	0	0	1 (1), 10.0
Dizziness	0	0	0	0	0	0	1 (1), 10.0
Skin and subcutaneous tissue disorders	0	0	0	1 (1), 16.7	1 (1), 16.7	2 (2), 6.7	0
Rash	0	0	0	1 (1), 16.7	1 (1), 16.7	2 (2), 6.7	0

a The data are shown as the number of subjects (number of events), percentage of subjects with adverse events. Denominators for the percentages are the numbers of subjects in the safety population in each treatment group.

**TABLE 3:** Incidence of adverse events in the multiple-dose study^*c*^

AE by system organ class, preferred term	Cefiderocol				Placebo (*n* = 10)
	1,000 mg 1st (*n* = 8)^*a*^	1,000 mg 2nd (*n* = 8)^*b*^	2,000 mg (*n* = 8)^*b*^	Total (*n* = 16)^*b*^	
Any adverse event	7 (17), 87.5	6 (15), 75.0	6 (13), 75.0	12 (28), 75.0	4 (6), 66.7
Gastrointestinal disorders	0	2 (3), 25.0	1 (1), 12.5	3 (4), 18.8	0
Diarrhea	0	2 (3), 25.0	0	2 (3), 12.5	0
Abdominal pain	0	0	1 (1), 12.5	1 (1), 6.3	0
General disorders and administration site conditions	2 (2), 25.0	1 (1), 12.5	1 (1), 12.5	2 (2), 12.5	0
Pyrexia	2 (2), 25.0	1 (1), 12.5	1 (1), 12.5	2 (2), 12.5	0
Infections and infestations	0	1 (1), 12.5	0	1 (1), 6.3	0
Upper respiratory tract infection	0	1 (1), 12.5	0	1 (1), 6.3	0
Investigations	5 (9), 62.5	4 (9), 50.0	4 (7), 50.0	8 (16), 50.0	4 (6), 66.7
Alanine aminotransferase level increased	1 (1), 12.5	3 (3), 37.5	1 (1), 12.5	4 (4), 25.0	3 (3), 50.0
Aspartate aminotransferase level increased	1 (1), 12.5	3 (3), 37.5	1 (1), 12.5	4 (4), 25.0	1 (1), 16.7
Blood creatine phosphokinase level increased	0	1 (2), 12.5	2 (2), 25.0	3 (4), 18.8	1 (1), 16.7
White blood cell count increased	0	0	2 (2), 25.0	2 (2), 12.5	0
Blood TSH level increased	3 (3), 37.5	0	0	0	0
Blood lactate dehydrogenase level increased	0	1 (1), 12.5	0	1 (1), 6.3	0
Blood urea level increased	1 (1), 12.5	0	0	0	1 (1), 16.7
Urine positive for white blood cells	0	0	1 (1), 12.5	1 (1), 6.3	0
Blood TSH level decreased	1 (2), 12.5	0	0	0	0
Blood present in urine	1 (1), 12.5	0	0	0	0
Nervous system disorders	1 (1), 12.5	0	1 (1), 12.5	1 (1), 6.3	0
Headache	1 (1), 12.5	0	1 (1), 12.5	1 (1), 6.3	0
Respiratory, thoracic, and mediastinal disorders	0	1 (1), 12.5	0	1 (1), 6.3	0
Oropharyngeal pain	0	1 (1), 12.5	0	1 (1), 6.3	0
Skin and subcutaneous tissue disorders	5 (5), 62.5	0	2 (3), 25.0	2 (3), 12.5	0
Rash	5 (5), 62.5	0	2 (3), 25.0	2 (3), 12.5	0

a The study drug (cefiderocol) was contaminated with iodide.

b The study drug (cefiderocol) was not contaminated with iodide.

c The data are shown as the number of subjects (number of events), percentage of subjects with adverse events. Denominators for the percentages are the numbers of subjects in the safety population in each treatment group.

In the single-dose study, nine adverse events (AEs) reported by six subjects in the cefiderocol groups were considered possibly or probably related to study treatment: diarrhea (two events in two subjects), rash (two events in two subjects), and one event each of abdominal pain, blood present in urine, urine positive for red blood cells, white blood cell count increased, and urine positive for white blood cells. In the placebo group, four AEs reported by three subjects were considered possibly related to study treatment (one event each of diarrhea, dizziness, nausea, and urine positive for white blood cells).

In the multiple-dose study, 16 AEs reported by seven subjects in the cefiderocol 1,000-mg 1st group were considered possibly or probably related to the study treatment: rash (5 events in five subjects), a blood thyroid-stimulating hormone (TSH) level increase (3 events in three subjects), pyrexia (2 events in two subjects), and 1 event each of an alanine aminotransferase level increase, an aspartate aminotransferase level increase, a blood TSH level decrease, a blood urea level increase, urine present in blood, and headache. In the cefiderocol 1,000-mg 2nd and 2,000-mg groups, 22 AEs reported by 12 subjects were considered possibly or probably related to the study treatment: an alanine aminotransferase level increase (4 events in four subjects), an aspartate aminotransferase level increase (3 events in three subjects), diarrhea (3 events in two subjects), rash (3 events in two subjects), pyrexia (2 events in two subjects), a white blood cell count increase (2 events in two subjects), and 1 event each of abdominal pain, a blood creatine phosphokinase level increase, headache, oropharyngeal pain, and urine positive for white blood cells. TSH abnormalities were reported only in the 1,000-mg 1st group.

In the multiple-dose study, there was a higher frequency of rash (five events in five of eight subjects [62.5%]) in the 1,000-mg 1st group than in the 1,000-mg 2nd (no events) and 2,000-mg groups (three events in two of eight subjects [25%]). Allergy tests conducted for the two subjects with rash in the 2,000-mg group showed levels almost within normal ranges, and measurement of cefiderocol-specific immunoglobulin G (IgG) and immunoglobulin E (IgE) yielded nondetectable levels.

All of the AEs were mild in intensity, except for one AE that was moderate (pyrexia in the 1,000-mg 1st group). There were no deaths, serious AEs, abnormal 12-lead or continuous electrocardiogram (ECG) findings, or abnormal changes in vital signs, except in the subjects in the multiple-dose study with pyrexia. One subject in the cefiderocol 1,000-mg 2nd group withdrew due to pyrexia on the last day of study drug administration. There was no dose-response trend in the incidence of AEs, which were relatively evenly spread among the dose groups, including the placebo groups.

### Blood iron and total iron-binding capacity levels.

In the single-dose study, the mean value of blood iron was slightly below the lower limit of normal (LLN) (reference range, 80 to 199 μg/dl for males, 70 to 179 μg/dl for females) in the 500-mg group on day 5 (71.2 μg/dl) and day 8 (68.3 μg/dl) and in the 1,000-mg group on day 8 (76.8 μg/dl); no mean value below the LLN was observed in the 2,000-mg group or the placebo group. In the multiple-dose study, the mean value of blood iron was slightly below the LLN in the 1,000-mg 1st group on days 5, 11, and 17 and in the 1,000-mg 2nd group on days 5, 11, 13, 14, and 17; no mean value below the LLN was observed in the 2,000-mg group or the placebo group (Table 4).

**TABLE 4:** Iron levels in multiple-dose study^*a*^

Time point	Statistic	Iron level (μg/dl)			
		Cefiderocol			Placebo (*n* = 6)
		1,000 mg 1st (*n* = 8)	1,000 mg 2nd (*n* = 8)^*b*^	2,000 mg (*n* = 8)	
Day 1 (baseline)	Mean (change from baseline)	91.6 (0)	96.5 (0)	132.3 (0)	118.7 (0)
	SD	17.2	23.3	49.9	32.2
	Median	90.5	101.5	127.5	103.0
	Range	59–115	50–129	60–230	102–183
Day 2	Mean (change from baseline)	82.5 (−9.1)	113.1 (+16.6)	123.6 (−8.6)	125.5 (+6.8)
	SD	22.3	17.1	35.6	21.9
	Median	86.5	113.5	126.0	119.5
	Range	52–116	80–135	81–196	100–164
Day 3	Mean (change from baseline)	95.5 (+3.9)	92.1 (−4.4)	111.6 (−20.6)	115.8 (−2.8)
	SD	17.8	15.7	35.5	29.8
	Median	91.5	93.0	103.0	115.5
	Range	72–119	69–114	60–182	84–161
Day 5	Mean (change from baseline)	74.5 (−17.1)	72.0 (−24.5)	109.1 (−23.1)	98.8 (−19.8)
	SD	13.1	18.4	24.8	16.0
	Median	72.5	68.0	104.5	106.5
	Range	55–94	53–104	86–166	77–113
Day 8	Mean (change from baseline)	96.9 (+5.3)	97.9 (+1.4)	103.4 (−28.9)	121.0 (+2.3)
	SD	19.1	12.6	21.2	23.5
	Median	95.5	103.0	106.5	113.5
	Range	64–130	81–110	74–143	95–156
Day 10	Mean (change from baseline)	80.5 (−11.1)	89.3 (−9.3)	89.3 (−43.0)	116.3 (−2.3)
	SD	25.7	18.0	33.4	27.3
	Median	87.0	100.0	92.0	122.5
	Range	23–104	65–105	52–154	78–152
Day 11	Mean (change from baseline)	65.0 (−26.6)	66.9 (−31.7)	95.8 (−36.5)	121.5 (+2.8)
	SD	26.6	36.9	36.1	21.7
	Median	77.5	41.0	102.5	130.5
	Range	14–87	35–116	46–141	82–138
Day 12	Mean (change from baseline)	86.6 (−5.0)	83.4 (−15.1)	109.1 (−23.1)	121.0 (+2.3)
	SD	30.6	22.9	40.2	22.2
	Median	98.0	84.0	114.5	121.0
	Range	18–111	55–111	51–168	91–156
Day 13	Mean (change from baseline)	87.1 (−4.5)	71.1 (−27.4)	90.4 (−41.9)	82.0 (−36.7)
	SD	41.6	25.7	13.4	48.1
	Median	77.5	63.0	90.5	67.5
	Range	50–178	36–107	75–116	39–167
Day 14	Mean (change from baseline)	95.6 (+4.0)	64.6 (−34.0)	85.4 (−46.9)	85.8 (−32.8)
	SD	32.9	26.0	27.8	33.5
	Median	93.5	65.0	74.5	77.5
	Range	56–154	29–109	57–136	51–137
Day 17	Mean (change from baseline)	60.6 (−31.0)	64.3 (−34.3)	100.9 (−31.4)	87.2 (−31.5)
	SD	33.3	17.5	33.9	32.8
	Median	48.5	69.0	103.5	76.0
	Range	33–139	37–82	59–168	49–128

a Reference range, 80 to 199 μg/dl for males and 70 to 179 μg/dl for females.

b *n* = 7 for days 10, 11, 12, 14, and 17.

### Pharmacokinetics in plasma.

**FIG 1:**
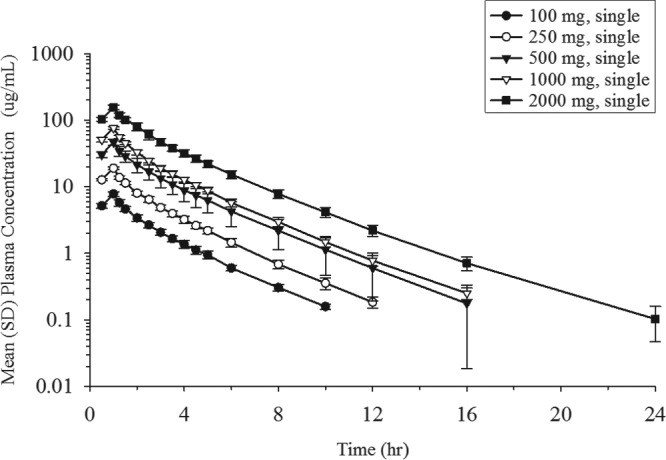
Mean (SD) plasma concentrations of cefiderocol following single-dose administration.

The mean plasma concentration profiles of unchanged cefiderocol after the infusion of single doses ranging from 100 to 2,000 mg are shown in Fig. 1. A summary of the values of the PK parameters following the single-dose administration of cefiderocol is shown in Table 5. Geometric mean values ranged from 7.76 to 156 μg/ml (coefficient of variation for geometric mean [CV], 4.6% to 10.7%) for the maximum plasma concentration (*C*_max_), 17.03 to 388.9 μg · h/ml (CV, 6.3% to 22.5%) for the area under the concentration-time curve (AUC) from time zero to the time of the last quantifiable concentration after dosing (AUC_0–last_), and 17.49 to 389.7 μg · h/ml (CV, 6.3% to 22.7%) for the area under the concentration-time curve extrapolated from time zero to infinity (AUC_0–inf_), suggesting low to moderate interindividual variability for plasma exposure in all dose groups. The geometric mean plasma terminal elimination half-life (*t*_1/2,*z*_) of cefiderocol was 1.98 to 2.74 h. Estimates of the slopes for the *C*_max_, AUC_0–last_, and AUC_0–inf_ of cefiderocol were 1.00 (95% confidence interval [CI], 0.965 to 1.04), 1.04 (95% CI, 0.983 to 1.09), and 1.03 (95% CI, 0.975 to 1.08), respectively, indicating dose-proportional increases in these parameters across the dose range of 100 to 2,000 mg. Statistical analyses showed no dose dependency for t_1/2,*z*_, total clearance (CL), the mean residence time (MRT), the urinary excretion ratio relative to the dose over 48 h (Feu_0–48_), or renal clearance (CL_R_).

**TABLE 5:** PK parameters for cefiderocol following single intravenous infusions of 100 to 2,000 mg^*a*^

PK parameter	Single-dose study, cefiderocol				
	100 mg (*n* = 6)	250 mg (*n* = 6)	500 mg (*n* = 6)	1,000 mg (*n* = 6)	2,000 mg (*n* = 6)
*C*_max_ (μg/ml)	7.76 (7.8)	18.9 (4.9)	46.6 (10.7)	76.4 (4.6)	156 (7.9)
*T*_max_ (h)	1.0 (1.0–1.0)	1.0 (1.0–1.0)	1.0 (1.0–1.0)	1.0 (1.0–1.0)	1.0 (1.0–1.0)
AUC_0–last_ (μg · h/ml)	17.03 (8.5)	41.41 (6.3)	108.0 (22.5)	167.3 (6.9)	388.9 (9.0)
AUC_0–inf_ (μg · h/ml)	17.49 (8.5)	41.94 (6.3)	108.6 (22.7)	168.1 (7.0)	389.7 (9.0)
*t*_1/2,*z*_ (h)	2.00 (4.4)	1.98 (5.5)	2.12 (15.5)	2.26 (5.8)	2.74 (10.2)
CL (liters/h)	5.72 (8.5)	5.96 (6.3)	4.60 (22.7)	5.95 (7.0)	5.13 (9.0)
MRT (h)	2.23 (3.9)	2.18 (6.2)	2.34 (15.2)	2.24 (4.4)	2.51 (4.7)
Feu_0–48_ (%)	68.4 (3.2)	64.0 (5.4)	65.8 (16.2)	68.3 (6.0)	61.5 (10.6)
CL_R_ (liters/h)	3.91 (8.8)	3.81 (10.7)	3.03 (38.3)	4.06 (11.2)	31.6 (16.8)

a The geometric mean (geometric mean percent coefficient of variation [CV]) is shown for all parameters except *T*_max_, for which the median (range) is shown. AUC_0–last_, area under the concentration-time curve from time zero to the time of the last quantifiable concentration after dosing; AUC_0–inf_, area under the concentration-time curve extrapolated from time zero to infinity; *C*_max_, maximum plasma concentration; CL, total clearance; CL_R_, renal clearance; Feu_0–48_, urinary excretion ratio relative to the dose over 48 h; MRT, mean residence time; PK, pharmacokinetics; *t*_1/2,*z*_, terminal elimination half-life; *T*_max_, time to the maximum plasma concentration.

**FIG 2:**
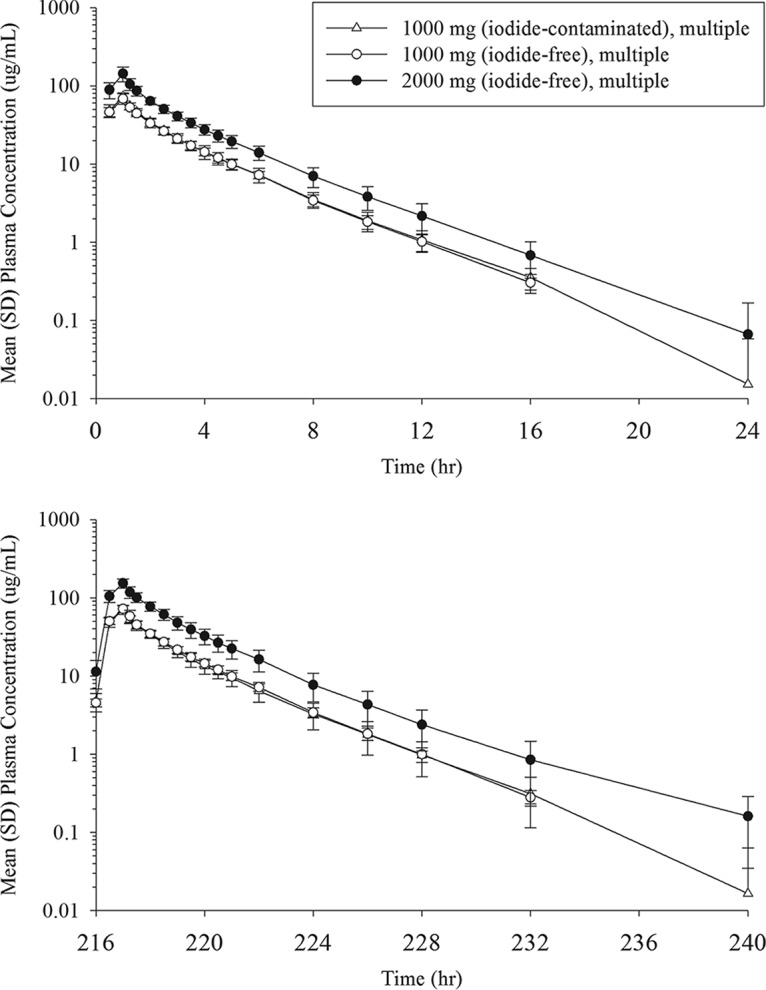
Mean (SD) plasma concentrations of cefiderocol following multiple-dose administration at 0 to 24 h (top) and 216 to 240 h (bottom) after the start of the initial infusion.

Figure 2 shows the mean plasma concentration profiles of cefiderocol following multiple infusions in the 1,000-mg 1st, 1,000-mg 2nd, and 2,000-mg groups. The mean trough concentrations from 48 to 192 h after the start of the initial infusion were 5.18 to 5.60, 5.11 to 5.39, and 9.66 to 13.0 μg/ml for the 1,000-mg 1st, 1,000-mg 2nd, and 2,000-mg groups, respectively. A summary of the values of the PK parameters for cefiderocol after multiple-dose administration is shown in Table 6. Plasma concentration profiles and linear regression for plasma trough concentrations indicated that steady state was achieved within 1 day after the initiation of multiple dosing (day 3). Plasma concentrations were similar in the 1,000-mg 1st and 1,000-mg 2nd groups. The ratios of *C*_max_ and the area under the concentration-time curve over dosing interval τ (AUC_0–τ_) between dose groups on day 10 were close to the dose ratio (i.e., 2), suggesting dose-proportional increases in *C*_max_ and AUC_0–τ_ following multiple dosing. Accumulation ratios of *C*_max_ and AUC with dosing every 8 h (q8h) were 1.069 and 1.053, respectively, at 1,000 mg and 1.084 and 1.164, respectively, at 2,000 mg. The comparisons of AUC (AUC_0–inf_ on day 1, AUC_0–τ_ on day 10), CL, and CL_R_ between days 1 and 10 indicated no change in PK with multiple dosing.

**TABLE 6:** PK parameters for cefiderocol following multiple intravenous infusions of 1,000 and 2,000 mg^*d*^

PK parameter	Multiple-dose study, cefiderocol					
	1,000 mg 1st (*n* = 8)		1,000 mg 2nd (*n* = 8)		2,000 mg (*n* = 8)	
	Day 1	Day 10	Day 1	Day 10	Day 1	Day 10
*C*_max_ (μg/ml)	72.2 (12.0)	69.8 (13.3)	68.1 (16.2)	72.2 (11.5)^*a*^	141 (22.7)	153 (12.9)
*T*_max_ (h)	1.0 (1.0–1.0)	1.0 (1.0–1.0)	1.00 (1.00–1.00)	1.00 (1.00–1.25)^*a*^	1.00 (1.00–1.25)	1.00 (1.00–1.25)
AUC_0–8_ (μg · h/ml)	165.5 (10.7)	NE	160.9 (10.5)	NE	314.8 (14.9)	NE
AUC_0–last_ (μg · h/ml)	176.4 (11.0)	NE	171.0 (10.6)	NE	337.2 (15.6)	NE
AUC_0–inf_ (μg · h/ml)	177.4 (10.9)	NE	172.0 (10.6)	NE	338.5 (15.5)	NE
AUC_0–τ_ (μg · h/ml)	NE	160.5 (13.5)	NE	168.6 (11.0)^*a*^	NE	366.5 (14.0)
*t*_1/2,_*_z_* (h)	2.37 (11.4)	2.35 (18.5)	2.25 (8.8)	2.19 (4.3)^*a*^	2.40 (13.2)	2.72 (21.6)
CL (liters/h)	5.64 (10.9)	6.23 (13.5)	5.81 (10.6)	5.93 (11.0)^*a*^	5.91 (15.5)	5.46 (14.0)
MRT (h)	2.49 (12.1)	NE	2.50 (6.8)	NE	2.53 (13.5)	NE
Feu^*c*^ (%)	70.9 (6.7)	70.0 (6.1)	63.8 (12.3)	64.7 (12.8)^*b*^	67.7 (4.7)	71.4 (5.3)
CL_R_ (liters/h)	4.02 (14.8)	4.36 (12.8)	3.73 (14.9)	3.85 (17.8)^*b*^	4.02 (17.2)	3.89 (15.1)

a *n* = 7.

b *n* = 6.

c Feu_0–24_ for day 1; Feu_0–8_ for day 10.

d The geometric mean (geometric mean percent coefficient of variation [CV]) is shown for all parameters except *T*_max_, for which the median (range) is shown. AUC_0–8_, area under the concentration-time curve from time zero to 8 h; AUC_0–last_, area under the concentration-time curve from time zero to the time of the last quantifiable concentration after dosing; AUC_0–inf_, area under the concentration-time curve extrapolated from time zero to infinity; AUC_0–τ_, area under the concentration-time curve over dosing interval τ; *C*_max_, maximum plasma concentration; CL, total clearance; CL_R_, renal clearance; Feu, urinary excretion ratio relative to the dose; MRT, mean residence time; NE, not evaluated; *t*_1/2_, terminal elimination half-life; *T*_max_, time to the maximum plasma concentration.

In the single-dose groups, the plasma concentrations of cefiderocol were below the lower limit of quantitation (BLQ) in most samples after 12 h at 100 mg, 16 h at 250 mg, 24 h at 500 and 1,000 mg, and 36 h at 2,000 mg. In the multiple-dose groups, the plasma cefiderocol concentration was BLQ in most samples after 24 h in both 1,000-mg groups and 36 h in the 2,000-mg group.

### Urinary excretion.

**FIG 3:**
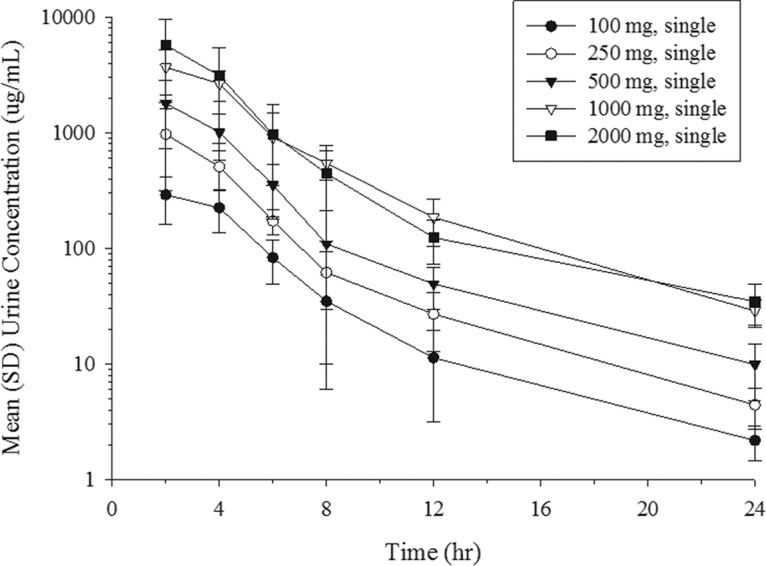
Mean (SD) urine concentrations of cefiderocol following single-dose administration.

**FIG 4:**
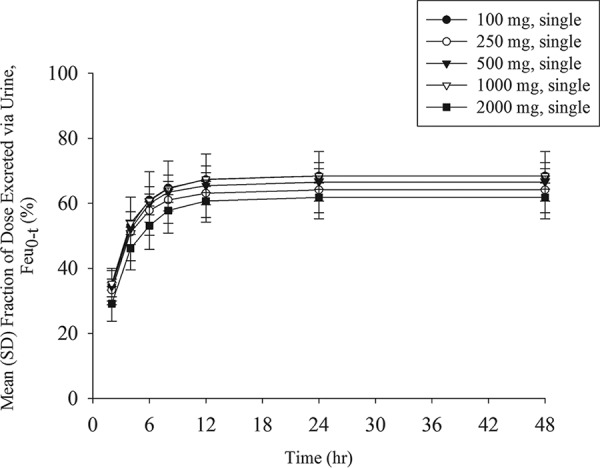
Mean (SD) fraction of cefiderocol dose excreted in urine following single-dose administration.

The mean urine concentration profiles of unchanged cefiderocol after the infusion of single doses ranging from 100 to 2,000 mg are shown in Fig. 3. Dose-dependent increases in the urine concentrations appeared across the dose range of 100 to 2,000 mg. The geometric mean urinary excretion ratio relative to the dose of cefiderocol ranged from 61.5% to 68.4% unchanged drug product (Fig. 4). The amount of cefiderocol metabolites was estimated to be less than 10%, as described below. The urine concentration of cefiderocol was BLQ in most urine samples collected from 24 to 48 h in all of the single-dose groups. In the multiple-dose groups, all urine samples had detectable levels of cefiderocol above the lower limit of quantitation at the nominal sampling time.

### Exploratory study of human metabolites in plasma and urine.

In plasma and urine, the most prominent peak identified by mass spectrometry (MS) was unchanged cefiderocol. Two types of methylated cefiderocol with a low MS response were detected, along with an additional nine metabolites. These metabolites had previously been identified in animal studies; there were no human-specific metabolites identified in plasma or urine. All urine metabolites were present at trace levels, and the total amount was 10% of the administered dose or less. Plasma metabolites showed no notable differences between single and multiple dosing, suggesting that the accumulation of metabolites is unlikely with multiple dosing.

## DISCUSSION

Cefiderocol is a novel parenteral siderophore cephalosporin with *in vitro* and *in vivo* efficacy against Gram-negative bacteria, including carbapenem-resistant Enterobacteriaceae, P. aeruginosa, and A. baumannii (9–11, 20). In the present study, cefiderocol was well tolerated at doses of up to 2,000 mg q8h in healthy volunteers. There were no serious or clinically significant AEs, and only one subject withdrew due to an AE (fever). The PK analysis showed dose-proportional increases in exposure at doses ranging from 100 to 2,000 mg, with little accumulation with multiple dosing. The majority of cefiderocol was excreted unchanged in the urine. The results from this study and a phase 1 single-dose study in subjects with renal impairment (17) were used to develop a population PK model for adjusting the dose of cefiderocol on the basis of renal function (18).

Because the proposed mechanism of action of cefiderocol involves iron chelation, the present study included laboratory tests to evaluate the effect of cefiderocol on iron homeostasis. Several multiple-dose cefiderocol groups had blood iron levels slightly below the lower limit of normal, which the investigator attributed to bone marrow iron uptake due to hematopoiesis from the frequent blood samplings. In our opinion, the fluctuation of the iron concentration in the blood was not clinically significant or related to the study drug. Further supporting our hypothesis, the total iron-binding capacity in the cefiderocol groups did not show a significant change from that in the placebo group (see Table S1 in the supplemental material).

With 60% to 70% of the administered dose of cefiderocol being excreted in the urine as unchanged parent drug and <10% being excreted as metabolites, the excretory fate of the remaining ∼20% is not known. Further study, such as a mass-balance trial, is needed to characterize the metabolism and excretion of cefiderocol.

Due to iodide contamination in the initial drug supply, additional safety measures were added to the multiple-dose study. The high frequency of rash and the TSH abnormalities in the 1,000-mg 1st group in the multiple-dose study were likely attributed to iodide exposure (19). As the formulation no longer contains iodide, these safety concerns are not relevant to the ongoing clinical development of cefiderocol.

In conclusion, this study indicates that single and multiple intravenous doses of cefiderocol are well tolerated in healthy subjects, and dose proportionality for the PK parameters was observed.

## MATERIALS AND METHODS

### Subjects.

Eligible subjects were healthy Japanese and Caucasian males or Japanese females not of childbearing potential (i.e., permanently sterilized or postmenopausal women) aged 20 to 60 years with body weights of 50.0 to 80.0 kg (40.0 to 80.0 kg for Japanese females, ≥50.0 kg for Caucasian males) and body mass indexes of ≥18.5 and <25.0 kg/m^2^ (≥18.5 and <30.0 kg/m^2^ for Caucasian males). Subjects with impaired heart, liver, renal, lung, endocrine, central nerve, blood, or metabolic function were excluded. All subjects provided written informed consent prior to the start of the study. The patients enrolled in the multiple-dose study were provided written informed consent that included additional literature-based safety information about iodide ingestion. The study was performed at the CPC Clinical Trial Hospital (Kagoshima, Japan) with the approval of the institutional ethics committee and in accordance with the principles of the Declaration of Helsinki and good clinical practice guidelines.

### Study design.

This was a phase 1, single-center, randomized, double-blind, placebo-controlled study conducted in two parts: a single-ascending-dose study and a multiple-ascending-dose study. The single-dose study was planned to include up to six single doses of cefiderocol in six cohorts (100, 250, 500, 1,000, 2,000, and 4,000 mg), but dose escalation to the highest dose (4,000 mg) was not performed because a lower dose achieved the prespecified stopping criteria exposure >10-fold lower than the no-observed-adverse-event level in rats (plasma concentration at the completion of dosing = 1,660 μg/ml). A total of 40 healthy Japanese males and females (6 individuals receiving the active drug and 2 individuals receiving a placebo per cohort) participated in the single-dose study, with 3, 1, and 1 postmenopausal females being used in the 500-mg, 1,000-mg, and 2,000-mg cohorts, respectively. Eligible subjects were admitted to the study center 1 day prior to administration, were confined to the study center until day 3, and returned to the study center on days 4, 5, and 8 ± 1 for follow-up.

The multiple-dose study was planned to include up to two doses of cefiderocol (1,000 and 2,000 mg) in two groups. It was discovered that the study drug was contaminated with 0.36% iodide (which is a level lower than the detection limit by traditional elemental analysis) before the start of the multiple-dose study. Consequently, a second 1,000-mg group with purified study drug was added to the multiple-dose study after the 1,000-mg 1st group finished the study. The study with the 1,000-mg 2nd group was conducted with study drug that was free of iodide, which was removed by additional purification. The study therefore included five groups that received study drug once at 100, 250, 500, 1,000, or 2,000 mg (which received iodide-contaminated study drug) and three groups that received the study drug every 8 h (q8h) for 10 days: the 1,000-mg 1st group (which received iodide-contaminated study drug), the 1,000-mg 2nd group (which received iodide-free study drug), and the 2,000-mg group (which received iodide-free study drug). A total of 30 healthy males (8 individuals receiving the active drug and 2 individuals receiving a placebo per cohort) participated in the multiple-dose study, with two Caucasians being included in each of the 1,000-mg-dose groups and three Caucasians being included in the 2,000-mg-dose groups. Eligible subjects were admitted to the study center 1 day prior to administration, were confined to the study center until day 12, and returned to the study center on days 13, 14, and 17 ± 1 for follow-up visits.

### Safety evaluation.

Safety was assessed through assessment of adverse events (AEs), physical examinations, assessment of vital signs, 12-lead and continuous electrocardiogram (ECG) recordings, and performance of laboratory tests (hematology; blood chemistry, including iron and total iron-binding capacity; and urinalysis). Thyroid function tests (tests for thyroid-stimulating hormone [TSH], free thyroxine, and free triiodothyronine levels) and allergy tests (tests for the blood tryptase level, serum complement activity, and the plasma histamine level) were added for the multiple-dose study in response to the iodide contamination in the initial drug supply, and additional immunological tests (tests for cefiderocol-specific immunoglobulin G [IgG] and immunoglobulin E [IgE]) were conducted for two subjects in the 2,000-mg cohort in the multiple-dose study who experienced a rash during the study. All safety data collected were assessed for severity and relationship to the study treatment. All AEs were coded using the Medical Dictionary for Regulatory Activities (MedDRA), version 15.0.

### Sample collection and analysis.

For the single-dose study, plasma samples were collected prior to infusion (0 h) and at 0.5 (during infusion), 1.0 (just before completion of infusion), 1.25, 1.5, 2.0, 2.5, 3.0, 3.5, 4.0, 4.5, 5.0, 6.0, 8.0, 10, 12, 16, 24, 36, and 48 h after the start of infusion. Urine samples were collected at 0 to 2, 2 to 4, 4 to 6, 6 to 8, 8 to 12, 12 to 24, and 24 to 48 h from the start of the infusion.

For the multiple-dose study, plasma samples were collected as follows: at 0, 0.5, 1.0, 1.25, 1.5, 2.0, 2.5, 3.0, 3.5, 4.0, 4.5, 5.0, 6.0, 8.0, 10, 12, and 16 h from the start of the first (morning) infusion on day 1; prior to the morning infusion (0 h) on days 2, 3, 5, 8, 9; and at 0, 0.5, 1.0, 1.25, 1.5, 2.0, 2.5, 3.0, 3.5, 4.0, 4.5, 5.0, 6.0, 8.0, 10, 12, 16, 24, 36, and 48 h from the start of the final (morning) infusion on day 10. Urine samples were collected at 0 to 8 and 8 to 24 h from the start of the first (morning) infusion on day 1 and at 0 to 8 and 8 to 24 h from the start of the final (morning) infusion on day 10.

The concentrations of cefiderocol in human plasma and urine were determined by a liquid chromatography (LC)-tandem mass spectrometry (MS/MS) method (17). The lower limit of quantification of cefiderocol was 0.1 μg/ml in plasma and 1 μg/ml in urine. Partial validation for changing instrumentation was performed using Qtrap 5500 and Triple Quad 5500 mass spectrometers. The assay was linear from 0.1 to 100 and 1 to 1,000 μg/ml for plasma and urine, respectively. The precision of the assay was 4.3% to 11.2% and 1.0% to 8.8% for plasma and urine, respectively. The accuracy of the assay was −7.0% to 7.0% and −6.4% to 9.0% for plasma and urine, respectively.

### Pharmacokinetic and statistical analyses.

The following PK parameters were calculated by noncompartmental methods using WinNonlin software (version 6.2.1; Certara L.P., Princeton, NJ) on the basis of plasma and urine concentration data: the maximum plasma concentration (*C*_max_), the time to the maximum plasma concentration (*T*_max_), the area under the concentration-time curve from time zero to the time of the last quantifiable concentration after dosing (AUC_0–last_), the area under the concentration-time curve extrapolated from time zero to infinity (AUC_0–inf_), the area under the concentration-time curve over dosing interval τ (AUC_0–τ_), the terminal elimination half-life (t_1/2,*z*_), the mean residence time (MRT), total clearance (CL), the urinary excretion ratio relative to the dose over 48 h (Feu_0–48_), and renal clearance (CL_R_).

The dose proportionality of the PK parameters was examined by using a power model. Dose proportionality, dose independency, the effect of multiple doses, and the accumulation ratio of PK parameters were examined by analysis of variance. Achievement of steady state was assessed by visual inspection of the plots and by linear regression for plasma trough concentrations. SAS software (version 9.1; SAS Institute Inc., Cary, NC) was used for statistical analyses.

### Exploratory study of human metabolites in plasma and urine.

Analysis and identification of cefiderocol and its metabolites were determined by use of an LC (Agilent 1100 series; Agilent Technologies)-MS/MS^n^ (LTQ Orbitrap Velos; Thermo Fisher Scientific) system. Exploratory investigations of major or human-specific minor metabolites were performed using pooled plasma samples (for the 2,000-mg-dose group in the single-dose study, 1, 2, 4, 12, and 24 h after dosing; for the 1,000-mg 1st and 2nd groups in the multiple-dose study, 1, 2, 4, 8, and 12 h after the morning dose on day 1 and 0, 1, 2, 4, and 8 h after the morning dose on day 10) and pooled urine (for the 2,000-mg-dose group in the single-dose study, 0 to 8, 8 to 12, 12 to 24, and 24 to 48 h after dosing).

## Supplementary Material

Supplemental material
