# Supplementary material for: Pharmacokinetics, Safety, and Tolerability of Cefiderocol, a Novel Siderophore Cephalosporin for Gram-Negative Bacteria, in Healthy Subjects
Source: Antimicrob Agents Chemother. 2018 Feb 23;62(3):e02163-17. doi: 10.1128/AAC.02163-17 (PMC5826143; doi:10.1128/AAC.02163-17)
Supplement: Supplemental material [file supp_62_3_e02163-17__index.html]

Supplemental material 

# Pharmacokinetics, Safety, and Tolerability of Cefiderocol, a Novel Siderophore Cephalosporin for Gram-Negative Bacteria, in Healthy Subjects

## Supplemental material

- Supplemental file 1 -

  Supplemental Table S1

  PDF, 67K
