# Supplementary material for: Pharmacokinetics, Safety, and Tolerability of Cefiderocol, a Novel Siderophore Cephalosporin for Gram-Negative Bacteria, in Healthy Subjects
Source: Antimicrob Agents Chemother. 2018 Feb 23;62(3):e02163-17. doi: 10.1128/AAC.02163-17 (PMC5826143; doi:10.1128/AAC.02163-17)
Supplement: Supplemental material [file AAC.02163-17_zac003186959s1.pdf]

**Online Supplement for:**

# Pharmacokinetics, Safety, and Tolerability of Cefiderocol, a Novel Siderophore Cephalosporin for Gram-Negative Bacteria, in Healthy Subjects

Yutaka Saisho[a]#, Takayuki Katsube[b], Scott White[c]*, Hiroyuki Fukase[d], Jingoro Shimada[e]

Medical Affairs Department, Shionogi & Co., Ltd., Osaka, Japan[a]; Clinical Research Department, Shionogi & Co., Ltd., Osaka, Japan[b]; Glaxo SmithKline, Philadelphia, PA, USA[c]; CPC Clinical Trial Hospital, Kagoshima, Japan[d]; Shionogi & Co., Ltd., Tokyo, Japan[e]

**Running Head:** Pharmacokinetics and Safety of Cefiderocol

#Address correspondence to Yutaka Saisho at yutaka.saisho@shionogi.co.jp

*Present address: Scott White, Clinical Development, Inovio Pharmaceuticals, Inc., Plymouth Meeting, PA, USA.

**Supplemental Table 1.** Total iron-binding capacity (μg/dl) in the multiple-dose study[a]

| | | Multiple-Dose Cefiderocol | | | |
|---|---|---|---|---|---|
| | | 1000 mg 1st | 1000 mg 2nd | 2000 mg | Placebo |
| Time point | Statistic | (n=8) | (n=8)[b] | (n=8) | (n=6) |
| Day 1 (baseline) | Mean (change from baseline) | 295.8 (0) | 283.1 (0) | 281.6 (0) | 332.7 (0) |
| | SD | 17.4 | 51.3 | 26.5 | 39.9 |
| | Median | 297.0 | 292.5 | 278.0 | 328.5 |
| | Range | 268–315 | 170–346 | 249–335 | 281–396 |
| Day 2 | Mean (change from baseline) | 296.0 (+0.3) | 279.0 (-4.1) | 294.6 (+13.0) | 328.3 (-4.3) |
| | SD | 15.4 | 52.8 | 27.7 | 36.0 |
| | Median | 302.0 | 285.5 | 287.5 | 322.5 |
| | Range | 266–314 | 162–339 | 266–349 | 280–384 |
| Day 3 | Mean (change from baseline) | 299.6 (+3.9) | 281.0 (-2.1) | 291.5 (+9.9) | 331.3 (-1.3) |
| | SD | 20.8 | 49.9 | 37.2 | 38.4 |
| | Median | 301.5 | 286.5 | 278.0 | 329.0 |
| | Range | 255–328 | 169–336 | 259–373 | 282–398 |
| Day 5 | Mean (change from baseline) | 295.6 (-0.1) | 273.1 (-10.0) | 290.1 (+8.5) | 338.7 (+6.0) |
| | SD | 23.3 | 47.3 | 35.3 | 30.2 |
| | Median | 302.0 | 279.5 | 285.5 | 328.0 |
| | Range | 249–317 | 170–334 | 245–358 | 304–379 |
| Day 8 | Mean (change from baseline) | 303.1 (+7.4) | 271.8 (-11.4) | 290.0 (+8.4) | 335.8 (+3.2) |
| | SD | 24.2 | 47.1 | 33.9 | 26.1 |
| | Median | 307.0 | 284.0 | 286.5 | 330.0 |
| | Range | 255–334 | 168–317 | 246–353 | 312–386 |
| Day 10 | Mean (change from baseline) | 291.4 (-4.4) | 287.4 (-11.9) | 277.8 (-3.9) | 321.8 (-10.8) |
| | SD | 25.4 | 22.1 | 35.4 | 28.6 |

| | | | | | |
|---|---|---|---|---|---|
| | Median | 294.5 | 276.0 | 271.0 | 312.5 |
| | Range | 248–322 | 265–321 | 234–354 | 296–376 |
| Day 11 | Mean (change from baseline) | 296.9 (+1.1) | 291.7 (-7.6) | 289.4 (+7.8) | 333.2 (+0.5) |
| | SD | 25.1 | 23.8 | 41.1 | 26.5 |
| | Median | 299.5 | 287.0 | 280.0 | 330.5 |
| | Range | 249–324 | 260–327 | 242–375 | 308–382 |
| Day 12 | Mean (change from baseline) | 303.8 (+8.0) | 301.1 (+1.9) | 298.3 (+16.6) | 334.5 (+1.8) |
| | SD | 21.4 | 17.6 | 47.1 | 28.9 |
| | Median | 303.0 | 300.0 | 291.0 | 330.5 |
| | Range | 270–337 | 281–329 | 250–385 | 309–388 |
| Day 13 | Mean (change from baseline) | 296.1 (+0.4) | 310.1 (+10.9) | 297.6 (+16.0) | 330.5 (-2.2) |
| | SD | 33.0 | 29.0 | 42.7 | 29.5 |
| | Median | 285.5 | 310.0 | 293.5 | 323.5 |
| | Range | 248–337 | 274–361 | 256–378 | 305–387 |
| Day 14 | Mean (change from baseline) | 290.8 (-5.0) | 290.0 (-9.3) | 292.1 (+10.5) | 323.5 (-9.2) |
| | SD | 26.1 | 27.6 | 31.3 | 25.7 |
| | Median | 285.5 | 294.0 | 289.0 | 314.5 |
| | Range | 252–333 | 260–332 | 255–341 | 296–367 |
| Day 17 | Mean (change from baseline) | 277.6 (-18.1) | 291.7 (-7.6) | 289.8 (+8.1) | 314.7 (-18.0) |
| | SD | 23.6 | 27.2 | 28.5 | 25.2 |
| | Median | 287.5 | 283.0 | 283.0 | 314.5 |
| | Range | 240–297 | 260–326 | 268–356 | 281–353 |

[a]Reference range: 290–355 μg/dl.

[b]n=7 for days 10, 11, 12, 14, and 17.
